# QTL Location and Epistatic Effect Analysis of 100-Seed Weight Using Wild Soybean (*Glycine soja* Sieb. & Zucc.) Chromosome Segment Substitution Lines

**DOI:** 10.1371/journal.pone.0149380

**Published:** 2016-03-02

**Authors:** Dawei Xin, Zhaoming Qi, Hongwei Jiang, Zhenbang Hu, Rongsheng Zhu, Jiahui Hu, Heyu Han, Guohua Hu, Chunyan Liu, Qingshan Chen

**Affiliations:** 1 Key Laboratory of Soybean Biology of Chinese Ministry of Education, Key Laboratory of Soybean Biology and Breeding/Genetics of Chinese Agriculture Ministry, College of Science, Northeast Agricultural University, Harbin, Heilongjiang Province, People’s Republic of China; 2 Land Reclamation Research & Breeding Centre of Heilongjiang, Harbin, Heilongjiang Province, People’s Republic of China; 3 School of Life Sciences and Center for Soybean Research of the Partner State Key Laboratory of Agrobiotechnology, The Chinese University of Hong Kong, Shatin, Hong Kong, SAR, China; Chinese Academy of Sciences, CHINA

## Abstract

Increasing the yield of soybean (*Glycine max* L. Merrill) is a main aim of soybean breeding. The 100-seed weight is a critical factor for soybean yield. To facilitate genetic analysis of quantitative traits and to improve the accuracy of marker-assisted breeding in soybean, a valuable mapping population consisting of 194 chromosome segment substitution lines (CSSLs) was developed. In these lines, different chromosomal segments of the Chinese cultivar Suinong 14 were substituted into the genetic background of wild soybean (*Glycine soja* Sieb. & Zucc.) ZYD00006. Based on these CSSLs, a genetic map covering the full genome was generated using 121 simple sequence repeat (SSR) markers. In the quantitative trait loci (QTL) analysis, twelve main effect QTLs (*qSW-B1*-1/2/3, *qSW-D1b*-1/2, *qSW-D2*-1/2, *qSW-G*-1/2/3, *qSW-M*-2 and *qSW-N*-2) underlying 100-seed weight were identified in 2011 and 2012. The epistatic effects of pairwise interactions between markers were analyzed in 2011 and 2012. The results clearly demonstrated that these CSSLs could be used to identify QTLs, and that an epistatic analysis was able to detect several sites with important epistatic effects on 100-seed weight. Thus, we identified loci that will be valuable for improving soybean 100-seed weight. These results provide a valuable foundation for identifying the precise location of genes of interest, and for designing cloning and marker-assisted selection breeding strategies targeting the 100-seed weight of soybean.

## Introduction

Soybean [*Glycine max* (L.) Merrill] is an important source of protein for humans and animals, and supplies more than half of the edible oil worldwide [1]. The 100-seed weight of soybean is a key determinant of seed yield. The 100-seed weight is often expressed as single seed weight, and it exhibits wide variation. The desirable seed size ranges from large for tofu, edamame, and miso, to small for natto production. In general, the soybean 100-seed weight ranges from 3.0 g to 77.5 g, but that of wild soybean (*Glycine soja* Sieb. & Zucc.) ranges from 0.50 g to 10.0 g [2]. The 100-seed weight is a quantitative trait, and is controlled by many genes with additive and epistatic effects [3, 4]. The construction of consensus linkage group maps for soybean had laid the foundation for analyses of QTLs (quantitative trait loci) that underlie important agronomic traits [5]. Many QTLs underlying the seed/100-seed weight have been identified [6–11]. Based on QTL location information, genome-wide association analyses have been performed using 257 soybean cultivars, seven wild soybean lines, and 302 wild and cultivar soybean cultivars, respectively [12–15]. Many QTLs and genome regions have been identified, and most of these sites have been mapped to the 20 soybean chromosomes in SoyBase (www.soybase.org). However, the molecular cloning of genes related to seed size/weight from soybean has lagged behind those of *Arabidopsis* and rice, because of the complex genomic structure of soybean and limited availability of genome sequence information.

The genetic basis of seed weight remains unclear; therefore, researchers have used a gene-mining strategy in wild species to identify genes related to seed weight in tomato and rice [16,17]. Genome-wide chromosome segment substitution line (CSSL) libraries represent marker-defined genomic regions taken from wild species and introgressed into the background of elite crop lines. The CSSLs genetic background is essential for the detection, validation, and positional cloning of QTLs combining data from genome [18,19]. A number of genes have been mined from wild genetic background germplasms in rice, maize, and tomato [20–26]. Combining QTL location data and genome data is an efficient strategy to clone a gene of interest. A number of QTLs have been found using different genetic populations of soybean. Some studies have used F_2_ and individual recombinant inbred lines (RILs) and multiple environments [12, 13]. However, many of the QTL loci could not be detected consistently in different years and different environments. This is because the genetic variation in complex traits includes both epistatic effects and environment × genotype interaction effects. Several QTLs with/without environmental and epistatic effects have been identified in recent years [16,27–29].

The genetic architecture of complex traits includes not only the actions of genes encoded by a single locus, but also inter-locus interactions. Compared with QTL × environment (QE) interactions, epistatic interactions have stronger effects on heterosis, inbreeding depression, adaptation, reproductive isolation, and speciation [30]. In this study, we identified and located the QTL underlying 100-seed weight of soybean, and analyzed the epsitatic effects of different pairwise marker interactions on 100-seed weight.

## Materials and Methods

### Genetic population and phenotypic evaluation of 100-seed weight

**Fig 1:**
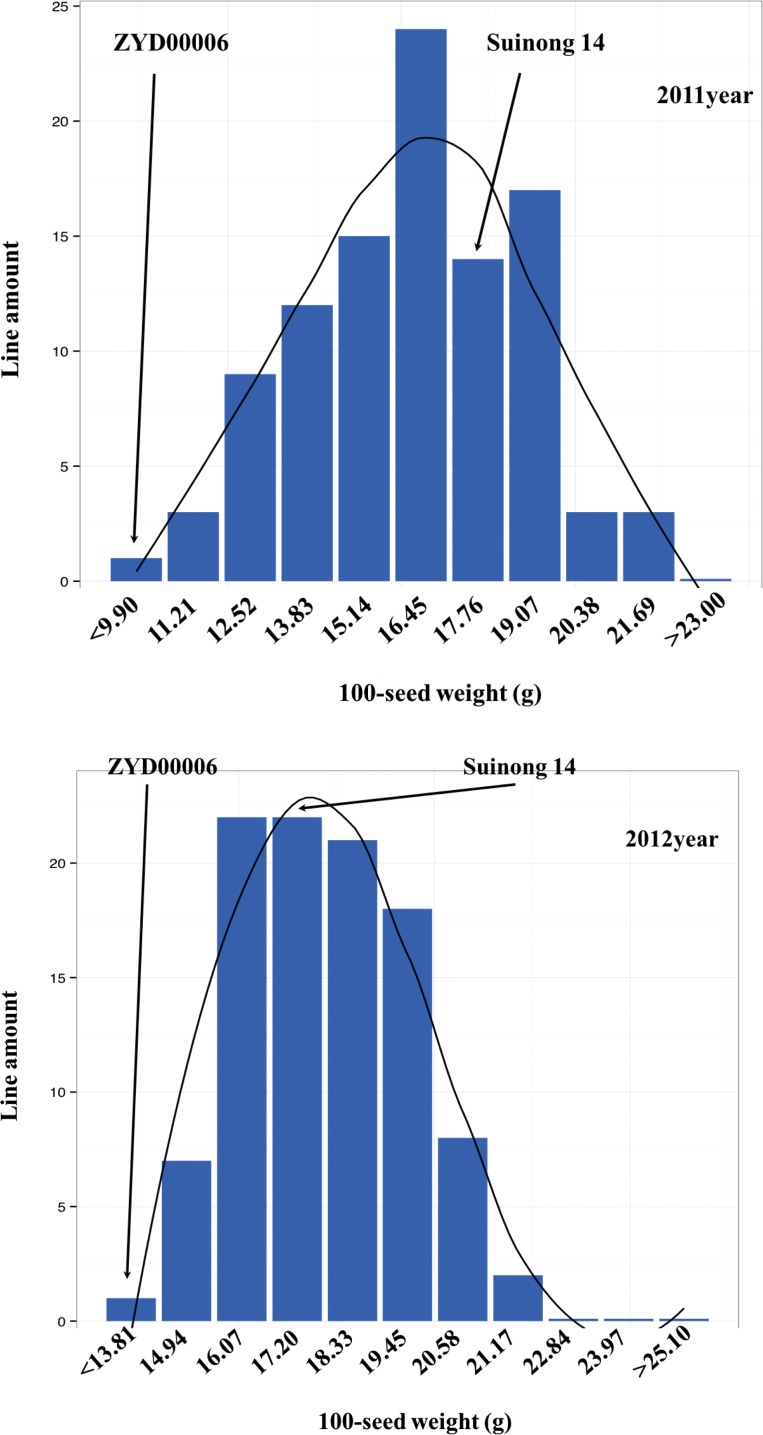
Frequency distribution of 100-seed weight in introgression lines.

A wild soybean CSSLs including 194 lines was constructed in this study. In 2012, 165 CSSL were obtained based one the 85 elementary CSSL by the marker selection, which including 190 substituted segment covered 82.55% genome of wild soybean. A fine mapping population was created by selecting homozygous recombinant BC_3_F_2_, BC_3_F_3_, BC_3_F_4_, BC_3_F_5_, and BC_3_F_6_ lines (S3 Fig). These lines were developed by backcrossing selected F_1_ lines (a cross between Suinong 14 as the recurrent parent and ZYD00006 as the donor parent) to Suinong 14 and then self-pollinating until the F_2_ generation. The 100-seed weight of Suinong 14 and ZYD00006 is 17.5 g and 2.57 g, respectively (Fig 1). By marker-assisted selection (MAS) for recurrent parent genome (S1 and S2 Figs; S2 and S3 Tables) and backcrossing again with Suinong 14, we obtained 85 BC_3_F_2_ lines. After several rounds of selfing and MAS, we obtained a total of 194 lines consisting of BC_3_F_2_, BC_3_F_3_, BC_3_F_4_, BC_3_F_5_, and BC_3_F_6_ individuals. The CSSLs and their parents were grown in a randomized complete block design with three replications in Harbin, China (45.75°N, 126.53°E) in 2011 and 2012. Seeds were planted with rows 5 m long, 0.65 m row spacing and with a space of 5 cm between two plants. Three replicates were used with a randomized complete block design, and fertilized with N 50kg/hm^2^, P_2_O 45kg/hm^2^ and K_2_O 25 kg/hm^2^. Value of 100-Seed weight was an average of five measurements of 100 randomly selected normal seeds.

### Marker development

Genomic DNA was extracted from leaf tissues of the two parents and the 194 CSSLs by the cetyl-trimethyl-ammonium bromide method [27]. In total, 329 simple sequence repeat (SSR) markers were used to screen for polymorphisms between the two parents. We used 121 SSR markers that were evenly distributed among the chromosomes to construct the linkage map with the CSSLs population. The size of the target chromosomal segment substituted from ZYD00006 into CSSLs ranged from a minimum of 0.22 cM to a maximum of 41.18 cM, and averaged 10.02 cM (S4 Fig). For the polymerase chain reaction (PCR), an optimal system in a 10 μL reaction volume was performed as Panaud et al [26] with minor modifications. The PCR system containing 1.0 μL of 10 × PCR Buffer (100 mmol/L Tris–HCl, pH 8.0; 500 mmol/L KCl; 0.1% gelatin), 1.0 μL of 2.5 mmol/L MgCl_2_, 0.20μL of 10mol/μL dNTP, 0.10 μL of Taq polymerase (5U/μL), 1.0 μL of each primer (100 μmol/L), 50 ng of genomic DNA, with the ultrapure grade water added to a total volume of 10 μL. The PCR reaction was operated in PTC-225 thermal cycler (MJ Research, Watertown, MA), and the condition of PCR was as follows: 95°C for 5 min, followed by 30 cycles of PCR amplification (94°C for 30 s, 55°C for 30 s, 72°C for 40 s) and a final extension at 72°C for 10 min. PCR products were kept at 4°C and detected polymorphism on 8% PAGE gel. Gels were run in PAGE running buffer (1×TBE) at 200 V for 2.0 h and then silver-stained.

### Statistical analyses and QTL detection

Based on data collected from the CSSL population in 2011 and 2012, a single factor analysis of 100-seed weight with SSR markers was performed using SPSS software [31]. QTL analysis was carried out using IciMapping software [5]. A threshold limit of detection (LOD) of 2.5 was applied to detect QTLs in non-ideal or ideal CSSLs populations (http://www.isbreeding.net/). Thresholds for QTL detection were set as P = 0.05 and were calculated by using 1,000 permutations at 1 cM interval. The length of substituted segment was calculated as the methods of Young [32] and Paterson [33]. Ignoring the two adjacent sections labeled double crossover occurred, when the genotype of an interval on both sides of the genome marker genotypes showed as donor (DD), it was considered as this interval segment was donor genotype. When the genome of an interval on side marker genotypes showed recipient genotype (RR), it was considered as the interval without donor genotype. When one side marker of an interval was labeled as donor genotypes, another side was recipient genotype, it was considered as this interval containing 50.0% of donor fragments (S5 Fig). Zone between the donor D1D2 segment substituted is the shortest length, then the length R1R2 is the maximum length, L is the estimate of the length of the substituted segments.

introduced the mean estimate for the length of the fragments minimum length and maximum length.

A substitution mapping approach was used to identify the exact position of the QTL, as described elsewhere [34]. The following equation was used to represent the genetic model for the QTL epistatic analysis, which modified from the model of Eshed and Zamire [34, 35]:

Y=p+SLHa+SLHb+SLHa×SLHb+ε

where *SLHa* and *SLHb* is the additive effect of introgression segment *a* in CSSL *a*, and introgression segment *b* in CSSL *b*, when segment *a* or segment *b* is present or absent, respectively, and SLHa*SLHb is the epistatic effect between segment *a* and segment *b*.

To increase the reliability of the epistatic effect analysis, the average value of the CSSL without segment *a* and *b* and Suinong 14 was used as the control genetic background when calculating the epistatic effect.

If Suinong 14 is the control, then

Y=(Suinong14+DSSLab)−SSSLa+SSSLb

If the CSSL without segment *a* and *b* is the control, then

Y=(LAA+DSSLab)−(SSSLa+SSSLb)

where LAA represents the average value with no target introgression segment, and DSSLab is the CSSL containing segment *a* and *b* at the same time. A significance level of P<0.05 was set as the threshold for the existence of an epistatic effect between segment *a* and segment *b*. MATLAB software was used to run these procedures (S1 Text). The QTL nomenclature followed the recommendations of McCouch [36].

## Results

### 100-seed weight phenotype

Table 1 shows the trait-wise mean comparison among Suinong 14, ZYD00006, and the CSSL population. There were significant variations in the 100-seed weight between 2011 and 2012 for Suinong 14, ZYD00006, and the CSSL population. The skew values were less than 1.0 in 2011 and 2012. And the Kurtosis value was negative in 2011 and more than 1.0 in 2012, respectively. These results suggesting that the segregation of the 100-seed weight phenotype was normally distributed (Table 1, Fig 1).

**Table 1:** Mean 100-seed weights of parent and CSSLs in 2011 and 2012.

Year	Trait	Parents		CSSL population						
		Suinong14	ZYD00006	Max	Min	Mean	Variance	StdError	Kurtosis	Skewness
2011	100-SW (g)	17.05	2.65	23.00	9.09	15.77	6.85	2.62	−0.23	0.05
2012	100-SW (g)	17.98	2.86	25.10	13.81	17.32	3.43	1.85	1.61	0.68

### SSR analysis by ANOVA

An ANOVA was performed in the SPSS software, which revealed that 16 SSR markers were related to 100-seed weight in 2011. These SSR markers were distributed on linkage groups (LGs) B1, C1, D1b, D2, F, G, H, J, M, and N. The LGs B1 and G had the most markers (three markers each). In 2012, 26 SSR markers were related to 100-seed weight. These SSR markers were distributed on LGs B1, C1, D1a, D1b, D2, F, G, I, K, M and N. The LGs F, G, I, M, and N all had three markers (Table 2). The SSR markers related to 100-seed weight in both 2011 and 2012 were Sat_261, Sat_149, Satt565, Sat_227, Satt594, Satt504, Satt505, Satt636, Sct_195, and Satt152.

**Table 2:** SSR Markers related to 100-seed weight in 2011 and 2012.

Linkage group	Locus	F value		Linkage group	Locus	F value	
		2011	2012			2011	2012
B1	Sat_261	4.52*	4.97*	G	Satt594	4.33*	7.52**
	Satt197	3.12*			Satt504	4.90**	6.95**
	Sat_149	5.67*	5.37*		Satt505	3.09*	5.51**
C1	Satt565	7.07**	3.69*	H	Satt568	5.35**	
D1a	Satt468		5.61**	I	Satt419		6.27*
	Satt147		10.61**		Satt671		3.20*
D1b	Sat_279		3.57*		Sat_324		4.89*
	Sat_227	6.09**	6.90**	J	Satt674	3.37*	
	Satg001		4.64*	K	Satt242		5.24*
D2	Satt582	4.22*		M	Satt636	5.93**	8.28*
	Satt669	3.65*			Satt567		3.39*
	Sat_001		5.26**		Sat_256		21.23**
	Sat_220		7.49**	N	Sct_195	3.27*	3.70*
F	Satt146	4.17*			Satt152	4.32*	4.93*
	Satt425		4.14*		Sat_306		3.32*
	Sat_317		10.89**				
	Satt554		6.27*				

*Significant at 0.05 level

** Significant at 0.01 level

### QTL mapping using CSSLs

Some neighboring SSR markers could not be identified at the same time due to the length of the substituted segment covered more than two SSR markers. This increased the probability of a negative relationship between SSR markers and phenotype. To avoid this bias, the difference in 100-seed weights between the recurrent parent and each CSSL with multiple markers in the same segment was analyzed by t-test (Table 3). The position of the QTL for 100-seed weight was identified in particular CSSLs by a substitution mapping approach.

**Table 3:** Significance of differences in 100-seed weight between CSSL lines and recurrent parent (t-test).

Material	100-seed weight±S (2011/2012)	Significanc (2011/2012)	Material	100-seed weight±S (2011/2012)	Significanc (2011/2012)
Suinong14	17.05±0.52/17.98±0.48		CSSL191	9.9±1.93/16.45±2.29	***/*
CSSL92	11.3±2.21/15.09±2.90	***/*	CSSL196	17.5±2.23/25.10±2.35	/***
CSSL93	12.67±2.34/16.32±3.02	***/	CSSL199	14.72±1.68/13.85±1.51	**/***
CSSL95	11.4±1.24/13.81±1.59	***/***	CSSL216	11.5±1.99/16.86±2.21	***/
CSSL102	13±1.88/14.79±1.72	***/***	CSSL220	12.36±0.78/15.72±0.70	***/***
CSSL103	12.4±1.12/14.95±1.25	***/***	CSSL224	12.51±1.56/15.52±1.72	***/**
CSSL117	14.1±1.43/17.86±1.13	***/	CSSL225	12.7±1.89/15.75±1.75	***/**
CSSL128	18.88±1.22/16.33±1.57	**/*	CSSL226	14.11±2.77/14.89±2.89	*/*
CSSL129	16.48±1.98/16.85±2.30	/	CSSL227	15.85±0.88/15.67±0.97	*/***
CSSL130	14.66±1.22/16.37±1.34	***/**	CSSL228	16.16±1.01/13.89±1.05	/***
CSSL145	15.33±0.56/15.40±0.79	***/***	CSSL239	15.53±1.22/18.83±1.57	*/
CSSL167	18.91±1.41/19.19±1.39	**/*	CSSL247	18.77±1.95/16.85±1.70	***/
CSSL169	16.91±1.29/19.13±1.31	/*	CSSL249	15.18±1.12/15.30±1.24	***/***
CSSL183	12.51+1.11/15.06±1.16	***/***	CSSL250	18.82±0.55/15.11±0.71	***/***
CSSL184	9.94±1.43/14.79±1.50	***/***	CSSL251	16.6±1.32/16.75±1.59	/*
CSSL189	12±1.56/14.93±1.65	***/***	CSSL259	13.78±0.55/16.02±0.00	***/***
CSSL190	15.85±2.12/15.66±1.98	/*			

* P≤0.05

** P≤0.01

*** P≤0.001

**Fig 2:**
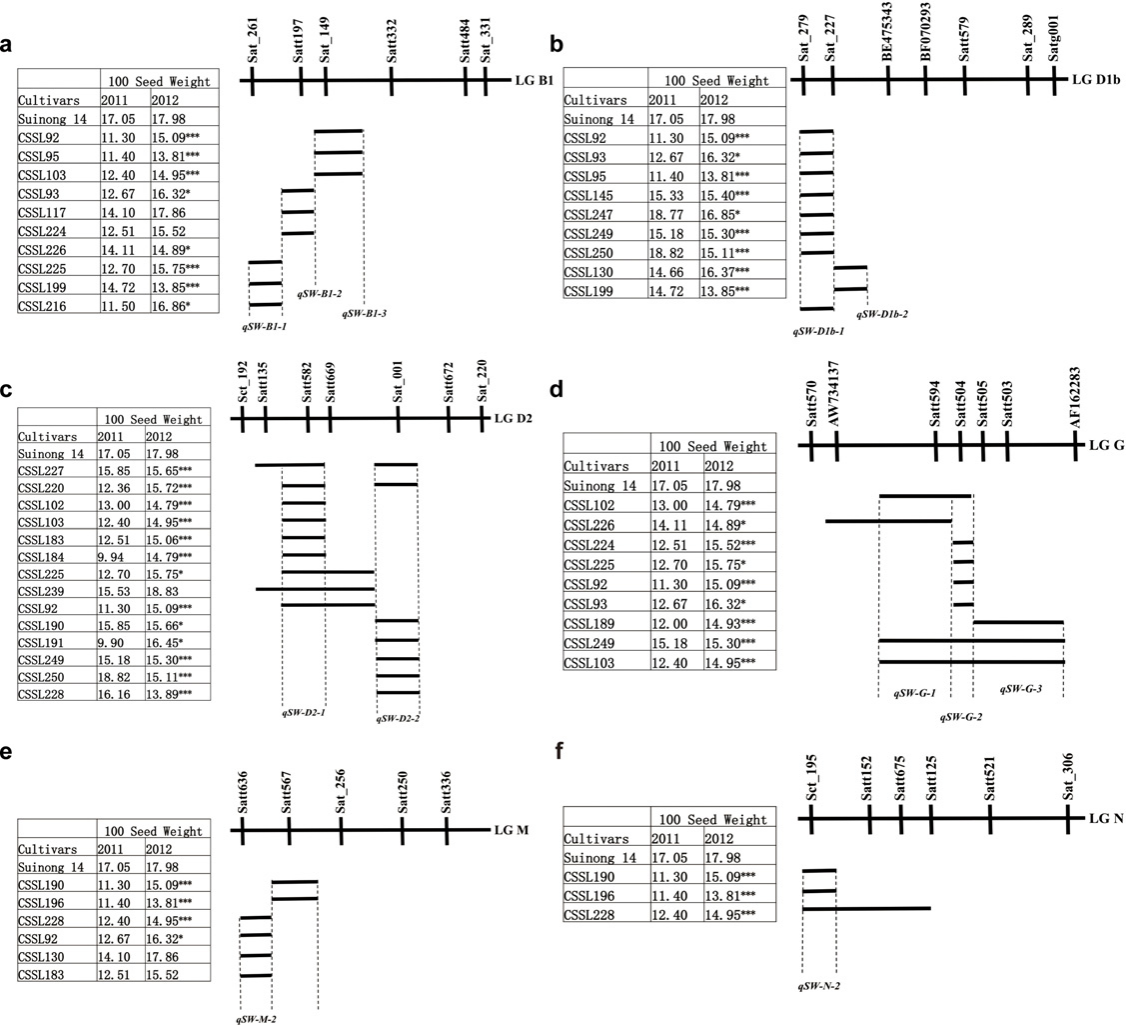
Substitution mapping of QTLs for 100-seed weight. a, b, c, d, e, f is substitution mapping of QTLs on linkage group B1, D1b, D2, G, M and N respectively.

The markers Sat_261, Satt197, and Sat_149 were identified on LG B1 (Fig 2A). Sat_261 was related to the QTL for 100-seed weight in both years (t-test). When this result was combined with substitution mapping, Sat_261 was found in three CSSL intervals that were significantly related to the 100-seed weight. Satt197 was only associated with the QTL in 2011 (t-test), and the 100-seed weight of CSSL117 (containing Satt197) was not significantly different from that of the recurrent parent. However, Sat_149 was identified in 2011 and 2012 (t-test), and this result was confirmed by substitution mapping.

Two neighboring SSR markers, Sat_279 and Sat_227, were identified on LG D1b by t-test (Fig 2B) in 2012. When this result was combined with substitution mapping, seven CSSLs containing Sat_279 showed significant relationships with the recurrent parent. Two CSSLs containing Sat_227 showed significant relationships with the recurrent parent.

Four neighboring SSR markers (Satt582, Satt669, Sat_001 and Sat_220) were identified on LG D2 by t-test in 2011 (Fig 2C). Satt582 was confirmed in nine CSSLs that showed significant relationships with the recurrent parent. However, one CSSL containing Satt582 did not have a significant relationship with the recurrent parent in 2012. Further research is required to locate the site of Satt582 more precisely. Satt669 was located in the overlap interval of Satt582, so the effect of Satt699 depended on Satt582. Satt699 was not a significant site for 100-seed weight. Seven CSSLs containing Sat_001 were identified by substitution mapping and Sat_001 was located on the overlap interval of seven CSSLs. These seven CSSLs all had significant relationships with the recurrent parent for 100-seed weight. No significant relationship was found in the CSSLs containing Sat_220

Three neighboring SSR markers (Satt594, Satt504, and Satt505) were identified on LG G by t-test in 2011 and 2012 (Fig 2D). When these results were combined with substitution mapping, Satt594 was located in the overlapping intervals of four CSSLs, Satt504 was located in the overlapping intervals of seven CSSLs, and Satt505 was located in the overlapping intervals of three CSSLs.

Three SSR markers (Satt636, Sat_256 and Satt567) were identified on LG M by t-test in 2011 and 2012 (Fig 2E). The location of Satt636 in the overlapping interval of four CSSLs was confirmed by substitution mapping. However, the location of Satt567 could not be confirmed by substitution mapping, and this marker had an inverse relationship with the recurrent parent in the t-test. These analyses showed that Satt567 was not related to the 100-seed weight.

Two neighboring SSR markers, Sct_195 and Satt152, were identified on LG N by t-test. Sct_195 was confirmed in three CSSLs by substitution mapping (Fig 2F). Satt152 was found in only one CSSL, which also contained Sct_195. Therefore, Satt152 was excluded from further analyses.

### Epistatic effect analysis of 100-seed weight in the Suinong 14 background

**Fig 3:**
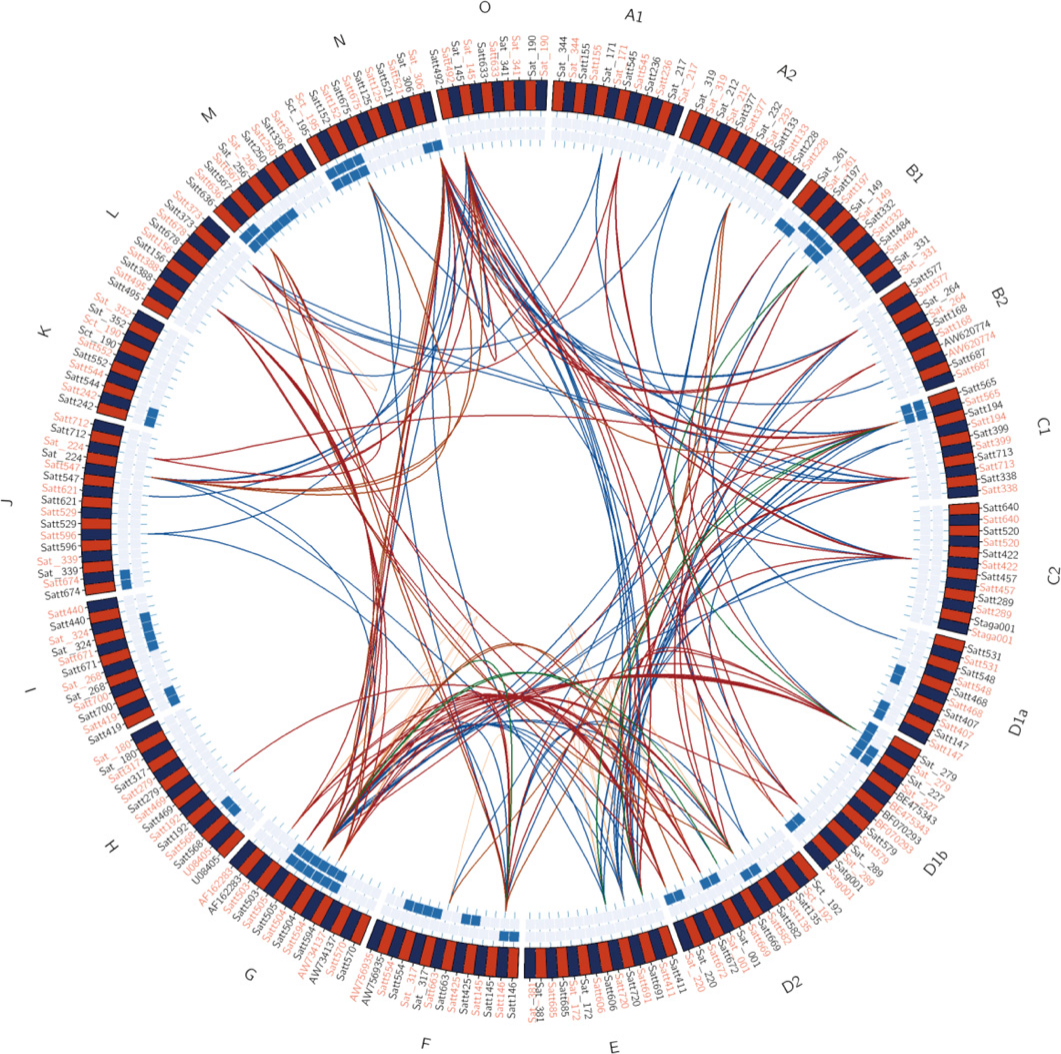
Distribution of epistatic loci about 100 seed weight on linkage groups. Note: contrast in 2011 and 2012; green and orange lines were detected with LAA as contrast in 2011 and 2012 year; blue box is related loci of 100 seed weight determined by analysis of variance in 2011 and 2012 year.

We conducted a combined genotype and phenotype analysis using 121 SSR markers and data from 2011 and 2012, and analyzed 7260 pairwise interactions by t-test (P≤0.05). In total, 111 pairwise interactions were detected in 2011 and 146 pairwise interactions were detected in 2012 (Fig 3, S1 Table).

Of the 111 pairwise interactions detected in 2011, 64 were identified as epistatic interactions by t-test (S1 and S5 Tables, Fig 3). Of the 64 epistatic interactions, 29 pairwise interactions had positive effects (ranging from 0.07% to 38.39%) and 35 pairwise interactions had negative effects (ranging from −34.47% to −0.07%). The pairwise interaction between AW620774 and Sat_306 had the strongest positive epistatic effect and that between Satt197 and Satt504 had the weakest positive epistatic effect. The pairwise interaction between Sat_261 and Satt531 had the strongest negative epistatic effect, and that between Satt504 and Satt547 had the weakest negative epistatic effect.

Of the 146 pairwise interactions detected in 2012, 68 were identified as epistatic interactions by t-test (S1 and S4 Tables, Fig 3). In total, 41 pairwise interactions had positive epistatic effects (ranging from 0.08% to 17.90%) and 27 pairwise interactions had negative epistatic effects (ranging from −35.25% to −0.10%). The pairwise interaction between Sat_001 and Satt594 had the strongest positive epistatic effect (17.90%) and the pairwise interaction between Satt504 and Satt503 had the weakest positive epistatic effect (0.08%). The pairwise interaction between Satt577 and Satt492 had the strongest negative epistatic effect (−35.25%) and that between Satt713 and Satg001 had the weakest negative epistatic effect (−0.10%).

### Epistatic effect analysis of 100-seed weight under the LAA background

When the CSSL without the target segment was used as the background, 110 pairwise epistatic interactions were identified in 2011, and 201 pairwise epistatic interactions were identified in 2012.

In 2011, epistatic effects were confirmed in 6 of 110 pairwise interaction sites by t-test (Table 4, Fig 3). Two pairwise interactions had positive effects (8.97% and 29.41%) and four pairwise interactions had negative effects (ranging from −28.49% to −4.50%). The pairwise interaction between Satt565 and Satt411 had the strongest positive epistatic effect, and that between Satt565 and Satt720 had the weakest positive epistatic effect. The pairwise interaction between Satt669 and Satt50 had the strongest negative epistatic effect and that between Sat_149 and Sat_279 had the weakest negative epistatic effect.

**Table 4:** Interacting loci related to 100-seed weight.

Year	Locus 1	Locus 2	Epistatic effect %	Year	Locus 1	Locus 2	Epistatic effect %
2011	Sat_149	Sat_279	−4.50	2012	Satt672	Satt504	11.82
	Satt565	Satt411	29.41		Sat_220	Satt504	12.58
	Satt565	Satt720	8.97		Satt146	Satt636	9.56
	Satt582	Satt504	−8.49		Satt594	Sat_306	8.29
	Satt669	Satt504	−28.49		Satt594	Satt504	11.47
	Satt146	Satt504	−7.51		Satt504	Sat_306	9.51
2012	Sat_232	Satt146	−16.32		Satt504	Satt503	−2.58
	Sat_232	Satt663	−13.57		Satt547	Satt152	9.18
	Satt565	Satt492	−30.82		Satt547	Sat_306	−18.04
	Satt582	Satt636	3.83		Satt547	Satt492	−20.40
	Satt672	Sat_220	−11.25				

In 2012, 15 of 201 pairwise epistatic interactions were confirmed by t-test (Table 4, Fig 3). Eight pairwise interactions had positive epistatic effects (ranging from 3.83% to 12.58%) and seven pairwise interactions had negative epistatic effects (ranging from −30.82% to −2.58%). The pairwise interaction between Sat_220 and Satt504 had the strongest positive epistatic effect (12.58%) and that between Satt582 and Satt636 had the weakest positive epistatic effect (3.83%). The pairwise interaction between Satt565 and Satt492 had the strongest negative epistatic effect (−30.82%) and that between Satt504 and Satt503 had the weakest negative epistatic effect (−2.58%).

### Comparison of interacting marker sites with epistatic effects between different years and different genetic backgrounds

**Fig 4:**
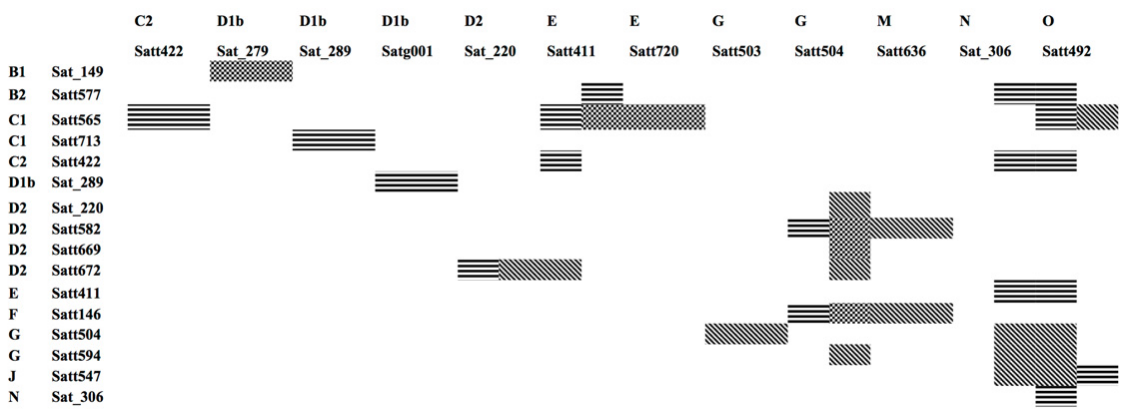
Comparison of epistatic loci in different years or contrasts. Note: Detected by both of contrasts at the same time in 2011 designated with a black gridding; Detected by both of contrasts at the same time in 2012 designated with a black diagonal line; Detected with Suinong 14 as contrast in 2011 and 2012 year designated with a black horizontal line; Detected with LAA as contrast in 2 years.

Under the Suinong 14 background, 20 pairwise interactions related to 100-seed weight were identified in both 2011 and 2012 (Table 5, Fig 4). These 20 pairwise interactions included 15 SSR marker sites: Sat_220, Sat_289, Sat_306, Satg001, Satt146, Satt411, Satt422, Satt492, Satt504, Satt547, Satt565, Satt577, Satt582, Satt672, and Satt713. Six pairwise interactions had opposite epistatic effects in 2011 and 2012 (Satt565 and Satt492, Satt565 and Satt422, Satt565 and Satt411, Satt422 and Satt411, Satt582 and Satt504, and Satt672 and Sat_220). These six pairwise interactions were sensitive to the environment. The other 13 pairwise interactions had similar epistatic effects in both years, so these interactions were insensitive to the environment. No stable pairwise interactions were identified in the control background (CSSL without the target segment).

**Table 5:** Pairwise interacting loci with epistatic effects on 100-seed weight in the Suinong 14 background in 2011 and 2012.

Locus 1	Locus 2	Epistatic effect %		Locus 1	Locus 2	Epistatic effect %	
		2011	2012			2011	2012
Satt577	Satt492	−20.92	−35.25	Satt422	Sat_306	−12.84	−8.93
Satt577	Satt411	−4.07	−15.97	Sat_289	Satg001	8.05	1.33
Satt577	Sat_306	−15.69	−6.85	Satt582	Satt504	−3.31	4.3
Satt565	Satt492	17.73	−26.46	Satt672	Sat_220	26.29	−8.59
Satt565	Satt422	1.45	−13.61	Satt411	Satt492	−9.83	−35.15
Satt565	Satt411	33.18	−6.06	Satt411	Sat_306	−3.9	−4.75
Satt565	Sat_306	2.61	0.37	Satt146	Satt504	−3.21	3.54
Satt713	Sat_289	7.71	5.78	Satt547	Sat_306	−20.49	−14.48
Satt422	Satt492	−13.7	−33.85	Satt547	Satt492	−14.14	−16.33
Satt422	Satt411	0.16	−19.32	Sat_306	Satt492	−19.35	−25.15

Six pairwise interactions were identified in 2011 under two backgrounds, and 11 pairwise interactions were identified in 2012 under two backgrounds (Table 6, Fig 4). These pairwise interactions were derived from 17 SSR markers.

**Table 6:** Interacting loci with epistatic effects on 100-seed weight in two genetic backgrounds in 2011 and 2012.

Year	Locus 1	Locus 2	Epistatic effect (%)		Year	Locus 1	Locus 2	Epistatic effect (%)	
			Suinong 14	LAA				Suinong 14	LAA
2011	Sat_149	Sat_279	1.66	−4.50	2012	Sat_220	Satt504	13.89	12.58
	Satt565	Satt411	33.18	29.41		Satt146	Satt636	11.21	9.56
	Satt565	Satt720	13.61	8.97		Satt594	Sat_306	9.84	8.29
	Satt582	Satt504	−3.31	−8.49		Satt594	Satt504	13.24	11.47
	Satt669	Satt504	−22.30	−28.49		Satt504	Sat_306	11.04	9.51
	Satt146	Satt504	−3.21	−7.51		Satt504	Satt503	0.08	−2.58
2012	Satt565	Satt492	−26.46	−30.82		Satt547	Sat_306	−14.48	−18.04
	Satt582	Satt636	5.14	3.83		Satt547	Satt492	−16.33	−20.40
	Satt672	Sat_220	−8.59	−11.25					

In 2011, two pairwise interaction sites had positive epistatic effects, three pairwise interaction sites had negative epistatic effects, and one pairwise interaction had opposite effects between the two genetic backgrounds. The pairwise interaction between Satt565 and Satt411 had the strongest positive epistatic effect (33.18% in Suinong 14 and 29.41% in LAA). The pairwise interaction between Satt669 and Satt504 had the strongest negative epistatic effect (−22.30% in Suinong 14 and −28.49% in LAA). The pairwise interaction between Sat_149 and Sat_279 had opposite epistatic effects under the two backgrounds (1.66% in Suinong 14 and -4.50% in LAA).

In 2012, six pairwise interactions had positive epistatic effects, four pairwise interactions had negative epistatic effects, and one pairwise interaction had opposite epistatic effects in the two genetic backgrounds. The pairwise interaction between Sat_220 and Satt504 had the strongest positive epistatic effect (13.89% in Suinong 14 and 12.58% in LAA). The pairwise interaction between Satt565 and Satt492 had the strongest negative epistatic effect (−26.46% in Suinong 14 and -30.82% in LAA). The pairwise interaction between Satt504 and Satt503 had opposite epistatic effects under the two genetic backgrounds (0.08% in Suinong 14 and −2.58% in LAA).

### Comparisons between QTLs and interacting loci with epistatic effects

We compared the pairwise interaction sites with the QTL for 100-seed weight under two backgrounds in 2 years. Five QTLs were consistent with 14 of 20 pairwise interaction sites in two years under the Suinong 14 background (Table 7). The QTL q*SW-C1* corresponded to Satt565 in four SSR marker combinations. These combinations had similar positive epistatic effects, and the additive effect of q*SW-C1* was negative in 2011. The QTL q*SW-N-2* corresponded with Sat_306 in six SSR marker combinations. The pairwise interaction between Sat_306 and Satt565 had a positive epistatic effect, while the other five pairwise interactions had negative epistatic effects. The QTL q*SW-G-2* corresponded with Satt504 in two SSR marker combinations with positive epistatic effects, but the additive effect of q*SW-G-2* was negative. The QTL *qSW-D1b-3* corresponded with Satg001 in one SSR marker combination with a positive epistatic effect, and the additive effect of *qSW-G-2* was negative. The QTL *qSW-D2-3* corresponded with Sat_220 in one SSR marker combinations with a negative epistatic effect, and the additive effect of *qSW-D2-3* was negative. None of the QTLs corresponded with particular markers in the LAA background.

**Table 7:** Comparison of epistatic interaction loci with 100-seed weight QTLs in 2011 and 2012.

Interaction loci		Epistatic effect (%)	QTL	Additive effect (%)	
				2011	2012
Satt565	Satt492	17.73	*qSW-C1*	−22.22	
Satt565	Satt422	1.45	*qSW-C1*	−22.22	
Satt565	Satt411	33.18	*qSW-C1*	−22.22	
Satt565	Sat_306	2.61	qSW-C1	−22.22	−11.56
Satt577	Sat_306	-6.85	*qSW-N-2*		−11.56
Satt565	Sat_306	0.37	*qSW-N-2*		−11.56
Satt422	Sat_306	-8.93	*qSW-N-2*		−11.56
Satt411	Sat_306	-4.75	*qSW-N-2*		−11.56
Satt547	Sat_306	-14.48	*qSW-N-2*		−11.56
Satt492	Sat_306	-25.15	*qSW-N-2*		−11.56
Satt146	Satt504	3.54	*qSW-G-2*		−14.58
Satt582	Satt504	4.3	*qSW-G-2*		−14.58
Sat_289	Satg001	1.33	*qSW-D1b-3*		−8.18
Satt672	Sat_220	-8.59	*qSW-D2-3*		−12.84

## Discussion

The lack of consistent QTL effects across different populations and different environments is one of the factors that limits their use for MAS [30]. In this study, the following pairs of markers were identified as stable epistatic interaction sites: Satt565 and Satt411, Satt582 and Satt504, Satt146 and Satt504, Satt672 and Sat_220, Satt565 and Satt492, Satt547 and Satt492. The identification of these sites provides a good foundation for MAS in breeding programs. The identification of stable QTLs can limit the effects of the environment on marker/trait associations. In previous studies, QTLs underlying yield, seed thickness, seed length to width, and seed width to thickness, were found near Satt565 in analyses of germplasms and two backcrossed populations [30, 37]. Also, Satt565 was found to be related to the number of branches on the main stem in analyses of wild CSSLs in soybean [37].

Satt504 was shown to be related to seed weight in an analysis of an F_2:3_ population of 186 families derived from a cross between Pak Chong 2 and Laos 7122 [38]. A QTL for the protein filling rate was found to be close to Satt504 [39]. Similarly, a QTL for seed shape was found to be close to Satt582, based on analyses of three densely mapped recombinant inbred populations, each with 192 segregants (Minsoy × Archer, Minsoy × Noir1, and Noir1 × Archer) [40]. The site of Satt146 was also located in analyses of three other RIL populations [41]. Satt672, Sat_220, and Satt547 were related to seed shape and seed weight in analyses of RILs and backcrossed populations in multiple environments [42, 43]. These results support that the location of the QTL identified in this study is reliable, and therefore, it could be used for further research on gene cloning and MAS breeding.

Main-effect QTLs are difficult to identify because of multi-gene effects. In such cases, the presence of epistasis may improve the detection of main-effect QTLs. If two epistatically interacting QTLs are misidentified as a main-effect QTL, the efficiency of MAS may be reduced [32–35, 37–44]. In this study, the sites of Satt504 and Satt492 were validated by the dissection of epistatic effects and identification of a single QTL. These two sites would not have been detected if we had only considered a main-effect QTL. We also found that some epistatic pairwise could be identified in two years, and some could not be found. This might due to the environment effect of some identified QTL. Multi-environment need to be involved in the application for the epistatic pairwise. Although we found the epistatic effect QTL underlying 100-seed weigh, to avoid the false-positive error. F_2_ population derived for a cross between QTL-CSSL and the recurrent parent should be constructed. Or by CSSL backcrossed to the recurrent parent to obtain a great number of secondary permanent mapping subpopulations. Combining with more marker selection in the target region, then the false-positive error could by avoided. More environments and years also need to be involved in the identification of epistatic QTL effect.

CSSLs are a valuable population for locating genes. Number of CSSL populations have been developed in rice, tomato, cotton, barley, wheat and maize, etc [34, 45–49]. It had been identified CSSL a powerful in precisely detecting and pyramiding genes/QTL/segments due to the clean background. In soybean there only few CSSL had been developed [45]. As an ideal CSSL population, single chromosome segment substitution line (SSSL) population which carrying only one different chromosome segment from the donor parent under the whole genome background of donor parent. In present study, the average recovery of the recurrent parent genome in this soybean CSSL was up to 90%, which could increase the confidence of QTL by reduce the background noise. In general, CSSLs could be used as a permanent mapping population [31], There are huge phenotypic difference between ZYD00006 and Suinong 14, so this CSSL would be very potential in mapping QTL for other wild traits. Some candidate QTL could be fine-mapped using F_2_ population derived from a cross between QTL-CSSL and the recurrent parent. By the development of CSSLs, CSSL could be cross with other CSSLs to study specific QTL by QTL interactions [50]. In this study, we found that some marker combinations had opposite epistatic effects, possibly because of genetic background noise. By increasing the amount of genomic data for soybean, more markers can be developed, for example, single nucleotide polymorphism (SNP) markers. Increasing the size of the CSSLs population, shortening the length of substituted segments, and reducing genetic background noise could result in more accurate location information for markers and genes of interest. Such information will lay the foundation for molecular studies to identify gene(s) of interest and their mode of action.

Two duplication events were followed by gene diversification and loss, and numerous chromosome rearrangements [51]. The location of QTL in this study might be located in the homoelogous regions. If the substituted fragment is short enough then some duplication could be avoided. Thus, these regions would need to be verified using additional markers to avoid missing unknown target segments. High-throughput genotyped CSSLs on the basis of whole-genome re-sequencing in soybean enabled the detection of numerous new segments compared to marker-based selection. It is necessary to purify the substituted chromosome to single substituted segment lines by reducing the residual non-target fragments from the donor in further study.

## Supporting Information

S1 Data Original data in Fig 1. (XLS)

S2 Data Original data in Fig 2. (XLS)

S3 Data Original data in Fig 3. (XLS)

S4 Data Original data in Fig 4. (XLS)

S1 Fig Genotypes of Genome-wide introgression line in 2012. (DOCX)

S2 Fig Genotypes of Genome-wide introgression line in 2013. (DOCX)

S3 Fig The population constructed procedure. (DOCX)

S4 Fig SSR markers distribution in Linkage Group. (DOCX)

S5 Fig Schematic diagram of substitution segment calculation. (DOCX)

S1 Table Percentage epistatic effect and epistatic loci of 100 seed weight in 2011 and 2012 years. (DOCX)

S2 Table Development of Genome-wide introgression line in 2012. (DOCX)

S3 Table Development of Genome-wide introgression lines in 2013. (DOCX)

S4 Table The significant pairwise interactions in 2012. (DOCX)

S5 Table The significant pairwise interactions in 2011. (DOCX)

S1 Text Matlab program for epistatic QTLs detected Annex 1 Matlab program for epistatic QTLs detected. (DOCX)
