# Supplementary material for: QTL Location and Epistatic Effect Analysis of 100-Seed Weight Using Wild Soybean (Glycine soja Sieb. & Zucc.) Chromosome Segment Substitution Lines
Source: PLoS One. 2016 Mar 2;11(3):e0149380. doi: 10.1371/journal.pone.0149380 (PMC4774989; doi:10.1371/journal.pone.0149380)
Supplement: S2 Fig — (DOCX) [file pone.0149380.s006.docx]

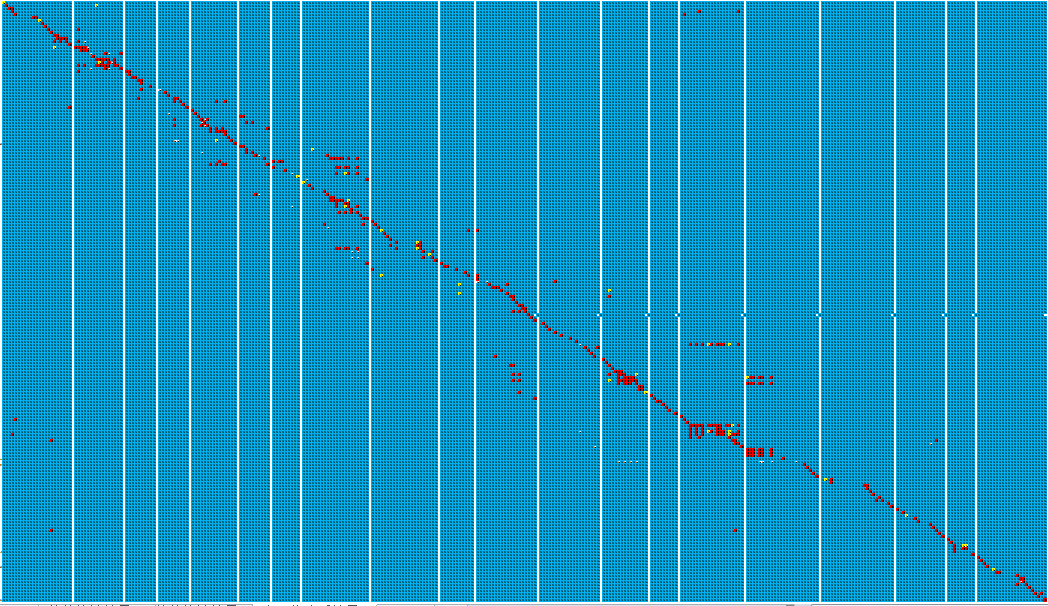


**注：** ZYD00006/Donor Suinong 14/Recurrent Heterozygous Unknow

Figure S2 Genotypes of Genome-wide introgression line in 20
