# Supplementary figures and images for: QTL Location and Epistatic Effect Analysis of 100-Seed Weight Using Wild Soybean (Glycine soja Sieb. & Zucc.) Chromosome Segment Substitution Lines
Source: PLoS One. 2016 Mar 2;11(3):e0149380. doi: 10.1371/journal.pone.0149380 (PMC4774989; doi:10.1371/journal.pone.0149380)

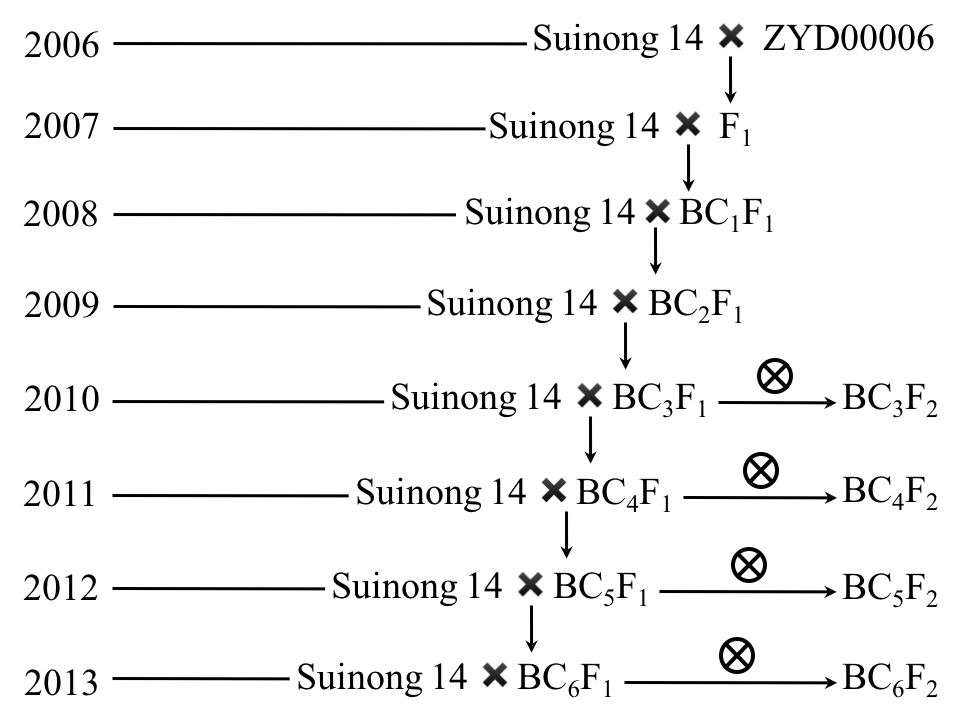


S3 fig. Thepopulation constructed procedure

Supplement: S3 Fig — (DOCX) [file pone.0149380.s007.docx]

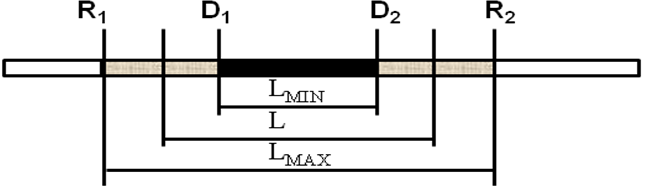


S5 fig. Schematic diagram of substitution segment calculation

Supplement: S5 Fig — (DOCX) [file pone.0149380.s009.docx]
