# Supplementary material for: QTL Location and Epistatic Effect Analysis of 100-Seed Weight Using Wild Soybean (Glycine soja Sieb. & Zucc.) Chromosome Segment Substitution Lines
Source: PLoS One. 2016 Mar 2;11(3):e0149380. doi: 10.1371/journal.pone.0149380 (PMC4774989; doi:10.1371/journal.pone.0149380)
Supplement: S1 Table — (DOCX) [file pone.0149380.s010.docx]

S1 Table Percentage epistatic effect and epistatic loci of 100 seed weight in 2011 and 2012 years

| 2011 | | | 2012 | | |
| --- | --- | --- | --- | --- | --- |
| Loci 1 | Loci 2 | Percentage of epistatic effect | Loci 1 | Loci 2 | Percentage of epistatic effect |
| Sat_261 | Satt531 | -34.47 | Satt577 | Satt492 | -35.25 |
| Satt596 | Satt152 | -22.96 | Satt411 | Satt492 | -35.15 |
| Satt669 | Satt504 | -22.30 | Satt422 | Satt492 | -33.85 |
| Satt577 | Satt492 | -20.92 | Satt565 | Satt492 | -26.46 |
| Satt547 | Sat_306 | -20.49 | Sat_306 | Satt492 | -25.15 |
| Sat_306 | Satt492 | -19.35 | Satt422 | Satt411 | -19.32 |
| Satt577 | Sat_306 | -15.69 | Satt691 | Satt720 | -18.38 |
| Satt547 | Satt492 | -14.14 | Satt547 | Satt492 | -16.33 |
| Satt422 | Satt492 | -13.70 | Satt577 | Satt411 | -15.97 |
| Satt135 | Satt152 | -13.42 | Satt503 | Satt388 | -15.40 |
| Satt422 | Sat_306 | -12.84 | Satt547 | Sat_306 | -14.48 |
| Satt720 | Satt492 | -12.48 | Satt565 | Satt422 | -13.61 |
| Sat_232 | Satt663 | -11.16 | Satt504 | Satt388 | -9.70 |
| Satt691 | Satt492 | -10.34 | Satt422 | Sat_306 | -8.93 |
| Satt411 | Satt492 | -9.83 | Satt672 | Sat_220 | -8.59 |
| Satt720 | Sat_306 | -9.65 | Satt545 | Satt713 | -7.85 |
| Satt713 | Satt373 | -7.72 | Satt505 | Satt388 | -7.14 |
| Satt691 | Sat_306 | -6.92 | Satt577 | Sat_306 | -6.85 |
| Satt577 | Satt691 | -6.64 | Satt594 | Satt388 | -6.77 |
| Sat_232 | Satt146 | -4.32 | Satt565 | Satt411 | -6.06 |
| Sat_171 | Satt621 | -4.07 | Satt545 | Satt373 | -5.73 |
| Satt577 | Satt411 | -4.07 | Satt411 | Sat_306 | -4.75 |
| Satt504 | Satt492 | -4.06 | Sat_220 | Satt388 | -3.58 |
| Satt411 | Sat_306 | -3.90 | Sat_001 | Satt388 | -1.02 |
| Satt577 | Satt504 | -3.71 | Sat_279 | Satt146 | -0.92 |
| Satt146 | Satt663 | -3.53 | Sat_279 | Satt503 | -0.64 |
| Satt582 | Satt504 | -3.31 | Satt713 | Satg001 | -0.10 |
| Satt146 | Satt504 | -3.21 | Satt504 | Satt503 | 0.08 |
| Satt691 | Satt547 | -2.48 | Satt168 | Satt146 | 0.09 |
| Sat_319 | Satt373 | -1.80 | Satt713 | Sat_224 | 0.24 |
| Satt135 | Satt146 | -1.63 | Satt545 | Satg001 | 0.37 |
| Satt422 | Satt691 | -0.76 | Satt565 | Sat_306 | 0.37 |
| Satt411 | Satt720 | -0.76 | Sat_224 | Satt373 | 0.69 |
| Satt582 | Satt146 | -0.57 | Sat_289 | Satg001 | 1.33 |
| Satt504 | Satt547 | -0.07 | Sat_279 | Sat_220 | 1.73 |
| Satt197 | Satt504 | 0.07 | Satt168 | Satt582 | 2.18 |
| Satt422 | Satt411 | 0.16 | Sat_149 | Satt504 | 2.22 |
| Satt422 | Satt504 | 0.82 | Sat_289 | Satt373 | 2.61 |
| Satt565 | Satt504 | 0.97 | Sat_001 | Satt672 | 2.72 |
| Satt194 | Satt720 | 1.26 | Sat_261 | Sat_279 | 2.72 |
| Satt565 | Satt422 | 1.45 | Satt146 | Satt594 | 3.00 |
| Sat_149 | Sat_279 | 1.66 | Satt146 | Satt504 | 3.54 |
| Satt720 | Satt504 | 1.94 | Satt545 | Sat_289 | 4.13 |
| Satt565 | Sat_306 | 2.61 | Satt582 | Satt504 | 4.30 |
| Satt194 | Satt691 | 3.40 | Satt594 | Satt503 | 4.43 |
| Sat_319 | Satt713 | 3.91 | Satt582 | Satt636 | 5.14 |
| Satt146 | Satt152 | 4.37 | Sat_279 | Satt504 | 5.38 |
| Satt691 | Satt504 | 4.66 | Satt582 | Satt594 | 5.54 |
| Satt146 | Satt596 | 4.85 | Sat_279 | Satt505 | 5.67 |
| Satt411 | Satt691 | 5.16 | Satt713 | Sat_289 | 5.78 |
| Satt713 | Sat_289 | 7.71 | Satt582 | Sat_306 | 5.87 |
| Sat_289 | Satg001 | 8.05 | Sat_220 | Satt503 | 5.93 |
| Satt411 | Satt504 | 8.24 | Satt582 | Satt672 | 5.96 |
| Sat_171 | Satt713 | 8.25 | Sat_149 | Satt582 | 6.05 |
| Satt713 | Satt678 | 8.27 | Sat_279 | Satt594 | 6.09 |
| Satt678 | Satt373 | 8.59 | Sat_001 | Satt503 | 7.20 |
| Satt194 | Satt504 | 9.68 | Sat_279 | Sat_001 | 8.04 |
| Satt565 | Satt720 | 13.61 | Sat_001 | Satt192 | 8.54 |
| Satt565 | Satt691 | 15.75 | Satt594 | Sat_306 | 9.84 |
| Satt565 | Satt492 | 17.73 | Satt504 | Sat_306 | 11.04 |
| Satt672 | Sat_220 | 26.29 | Satt146 | Satt636 | 11.21 |
| Satt388 | Sat_306 | 31.94 | Satt582 | Sat_001 | 11.69 |
| Satt565 | Satt411 | 33.18 | Satt594 | Satt504 | 13.24 |
| AW620774 | Sat_306 | 38.39 | Sat_220 | Satt504 | 13.89 |
|  |  |  | Sat_220 | Satt505 | 14.24 |
|  |  |  | Sat_220 | Satt594 | 14.60 |
|  |  |  | Sat_001 | Satt504 | 14.97 |
|  |  |  | Sat_001 | Satt505 | 17.49 |
