# Supplementary material for: QTL Location and Epistatic Effect Analysis of 100-Seed Weight Using Wild Soybean (Glycine soja Sieb. & Zucc.) Chromosome Segment Substitution Lines
Source: PLoS One. 2016 Mar 2;11(3):e0149380. doi: 10.1371/journal.pone.0149380 (PMC4774989; doi:10.1371/journal.pone.0149380)
Supplement: S2 Table — (DOCX) [file pone.0149380.s011.docx]

S2 Table Development of Genome-wide introgression line in 2012

| Linkage Group | Numbers of marker | Numbers of donor segments | Length of total donor segments(cM) | Average of donor segments(cM) | Longest segment  (cM) | Shortest segment (cM) | coverage% |
| --- | --- | --- | --- | --- | --- | --- | --- |
| A1 | 23 | 13 | 101.345 | 7.8 | 29.125 | 0.95 | 95.43 |
| A2 | 16 | 11 | 237.21 | 21.56 | 50.74 | 4.44 | 86.04 |
| B1 | 10 | 7 | 128.3 | 18.33 | 33.26 | 1.28 | 77.71 |
| B2 | 10 | 8 | 110.6 | 13.82 | 3.92 | 28.41 | 81.28 |
| C1 | 15 | 9 | 144.28 | 16.03 | 33.44 | 0.38 | 85.43 |
| C2 | 10 | 5 | 105.92 | 21.18 | 33.77 | 3.12 | 92.77 |
| D1a | 9 | 6 | 123.77 | 20.63 | 23.87 | 2.31 | 73.58 |
| D1b | 22 | 13 | 168.08 | 12.93 | 22.36 | 1.66 | 84.01 |
| D2 | 22 | 13 | 118.41 | 9.11 | 23.84 | 1.87 | 78.18 |
| E | 11 | 7 | 58.97 | 8.42 | 23.83 | 0.75 | 78.7 |
| F | 20 | 11 | 162.62 | 14.78 | 20.09 | 1.12 | 84.21 |
| G | 20 | 14 | 119.19 | 8.51 | 18.31 | 0.64 | 96.69 |
| H | 15 | 7 | 124.05 | 17.72 | 29.15 | 6.63 | 92.63 |
| I | 9 | 7 | 102.33 | 14.62 | 32.27 | 6.57 | 86.13 |
| J | 21 | 9 | 140.43 | 15.6 | 22.28 | 0.68 | 93.86 |
| K | 24 | 10 | 83.74 | 8.37 | 34.45 | 0.22 | 70.29 |
| L | 24 | 9 | 73.26 | 8.14 | 18.44 | 2.23 | 46.59 |
| M | 16 | 10 | 89.66 | 8.97 | 15.95 | 2.5 | 67 |
| N | 9 | 6 | 104.23 | 17.37 | 26.24 | 11.34 | 95.97 |
| O | 23 | 15 | 93.86 | 6.26 | 9.72 | 1.2 | 84.54 |
| Aveerage | 16.45 | 9.5 | 119.51 | 13.51 | 25.25 | 3.91 | 82.55 |
