# Supplementary material for: QTL Location and Epistatic Effect Analysis of 100-Seed Weight Using Wild Soybean (Glycine soja Sieb. & Zucc.) Chromosome Segment Substitution Lines
Source: PLoS One. 2016 Mar 2;11(3):e0149380. doi: 10.1371/journal.pone.0149380 (PMC4774989; doi:10.1371/journal.pone.0149380)
Supplement: S3 Table — (DOCX) [file pone.0149380.s012.docx]

S3 Table Development of Genome-wide introgression lines in 2013

| Linkage Group | Numbers of marker | | Numbers of donor segments | | Length of total donor segments (cM) | | | Average of donor segments  (cM) | | Longest segment  (cM) | | Shortest segment  (cM) | coverage% | |  |
| --- | --- | --- | --- | --- | --- | --- | --- | --- | --- | --- | --- | --- | --- | --- | --- |
| A1 | | 23 | | 15 | | 96.425 | 6.42 | | 17.72 | | 0.655 | | | 96.55 | |
| A2 | | 16 | | 7 | | 136.54 | 19.51 | | 41.18 | | 4.37 | | | 90.71 | |
| B1 | | 10 | | 6 | | 111.05 | 18.51 | | 26.02 | | 1.28 | | | 80.61 | |
| B2 | | 10 | | 6 | | 85.97 | 14.33 | | 20.2 | | 6.24 | | | 78.33 | |
| C1 | | 15 | | 12 | | 130.05 | 10.84 | | 36.66 | | 0.38 | | | 99.56 | |
| C2 | | 10 | | 6 | | 98.22 | 16.37 | | 33.77 | | 3.12 | | | 93.07 | |
| D1a | | 9 | | 4 | | 50.57 | 12.64 | | 23.87 | | 2.31 | | | 68.77 | |
| D1b | | 22 | | 13 | | 129.79 | 9.98 | | 22.36 | | 0.71 | | | 83.86 | |
| D2 | | 22 | | 14 | | 112.94 | 8.07 | | 16.81 | | 1.19 | | | 85.85 | |
| E | | 11 | | 5 | | 40.71 | 8.14 | | 13.98 | | 0.75 | | | 73.49 | |
| F | | 20 | | 14 | | 162.13 | 11.58 | | 18.4 | | 1.12 | | | 90.1 | |
| G | | 20 | | 11 | | 48.61 | 4.42 | | 15.52 | | 0.6 | | | 56.32 | |
| H | | 15 | | 12 | | 153.38 | 12.78 | | 15.82 | | 5.04 | | | 100 | |
| I | | 9 | | 7 | | 87.64 | 12.52 | | 17.58 | | 6.57 | | | 87.48 | |
| J | | 21 | | 11 | | 87.94 | 7.99 | | 13.16 | | 0.25 | | | 99.53 | |
| K | | 24 | | 9 | | 67.6 | 7.51 | | 34.45 | | 0.22 | | | 72.18 | |
| L | | 24 | | 11 | | 87 | 7.91 | | 18.44 | | 0.45 | | | 73.76 | |
| M | | 16 | | 10 | | 99.71 | 9.97 | | 17.8 | | 2.5 | | | 74.5 | |
| N | | 9 | | 5 | | 61.95 | 12.39 | | 16.04 | | 5.99 | | | 67.84 | |
| O | | 23 | | 16 | | 95.64 | 6.38 | | 14.64 | | 1.28 | | | 92.17 | |
| Average | | 16.45 | | 9.7 | | 97.19 | 10.91 | | 21.72 | | 2.25 | | | 83.23 | |
