# Supplementary material for: QTL Location and Epistatic Effect Analysis of 100-Seed Weight Using Wild Soybean (Glycine soja Sieb. & Zucc.) Chromosome Segment Substitution Lines
Source: PLoS One. 2016 Mar 2;11(3):e0149380. doi: 10.1371/journal.pone.0149380 (PMC4774989; doi:10.1371/journal.pone.0149380)
Supplement: S4 Table — (DOCX) [file pone.0149380.s013.docx]

S4 Table The significant pairwise interactions in 2012

| 2012 |  | 2012 |  | 2012 |  | 2012 |  | 2012 |  |
| --- | --- | --- | --- | --- | --- | --- | --- | --- | --- |
| Satt505 | Satt503 | Satt146 | Satt636 | Satt194 | Sat_306 | Sat_001 | Satt503 | Sat_261 | Satt135 |
| Satt594 | Satt504 | Satt146 | Satt504 | Satt194 | Satt492 | Sat_001 | Satt388 | Satt577 | Satt691 |
| Satt713 | Sat_289 | Satt425 | Satt663 | Satt422 | Satt411 | Sat_220 | Satt594 | Satt577 | Satt504 |
| Satt582 | Satt504 | Satt504 | Sat_306 | Satt422 | Sat_306 | Sat_220 | Satt505 | Satt565 | Satt691 |
| Satt547 | Sat_306 | Satt582 | Satt672 | Satt422 | Satt492 | Sat_220 | Satt503 | Satt565 | Satt720 |
| Satt594 | Satt505 | Sat_001 | Satt672 | Satt411 | Sat_306 | Sat_220 | Satt388 | Satt565 | Satt504 |
| Satt594 | Satt503 | Satt582 | Sat_001 | Satt411 | Satt492 | Satt594 | Satt388 | Satt194 | Satt691 |
| Satt504 | Satt505 | Satt425 | Satt152 | Satt547 | Satt492 | Satt504 | Satt388 | Satt194 | Satt720 |
| Satt504 | Satt503 | Sat_001 | Satt192 | Sat_306 | Satt492 | Satt505 | Satt388 | Satt194 | Satt504 |
| Satt545 | Sat_289 | Sat_279 | Satt146 | Satt582 | Sat_306 | Satt503 | Satt388 | Satt422 | Satt691 |
| Satt545 | Satg001 | Sat_289 | Sat_224 | Satt582 | Satt146 | Satt669 | Satt504 | Satt422 | Satt504 |
| Satt691 | Satt720 | Satg001 | Sat_224 | Sat_149 | Satt582 | Sat_279 | Satt672 | Satt411 | Satt720 |
| Sat_261 | Sat_279 | Satt577 | Satt194 | Sat_149 | Satt504 | Satt672 | Satt504 | Satt411 | Satt504 |
| Satt672 | Sat_220 | Satt577 | Satt411 | Satt582 | Satt594 | Sat_212 | Satt145 | Satt691 | Satt504 |
| Satt545 | Satt713 | Satt577 | Sat_306 | Satt146 | Satt594 | Satt145 | Satt152 | Satt691 | Sat_306 |
| Satt545 | Satt373 | Satt577 | Satt492 | Satt713 | Sat_224 | Satt594 | Sat_306 | Satt691 | Satt492 |
| Satt713 | Satg001 | Satt565 | Satt194 | Sat_224 | Satt373 | Sat_212 | Satt152 | Satt720 | Satt504 |
| Sat_289 | Satt373 | Satt565 | Satt422 | Sat_279 | Sat_001 | Sat_232 | Satt146 | Satt720 | Sat_306 |
| Sat_001 | Sat_220 | Satt565 | Satt411 | Sat_279 | Satt594 | Sat_232 | Satt663 | Satt720 | Satt492 |
| Sat_279 | Sat_220 | Satt565 | Sat_306 | Sat_279 | Satt505 | Satt146 | Satt663 | Satt504 | Satt547 |
| Sat_279 | Satt504 | Satt565 | Satt492 | Sat_279 | Satt503 | Sat_001 | Sat_306 | Satt504 | Satt492 |
| Sat_220 | Satt504 | Satt194 | Satt422 | Sat_001 | Satt594 | Sat_220 | Sat_306 | Satt168 | Satt582 |
| Sat_289 | Satg001 | Satt194 | Satt411 | Sat_001 | Satt504 | Satt135 | Sat_324 | Satt168 | Satt146 |
| Satt582 | Satt636 | Satt194 | Satt547 | Sat_001 | Satt505 | Sat_149 | Satt146 | Sat_261 | Satt531 |
| Sat_227 | Satt146 | Satt545 | Satt579 | Satt579 | Sat_289 | Satt577 | Satt565 | Satt570 | AW734137 |
| Sat_319 | Satt713 | Sat_319 | Satt579 | Satt579 | Satg001 | Satt565 | Satt547 | Satt504 | Satt440 |
| Sat_279 | Sat_324 | Sat_319 | Sat_289 | Satt579 | Satt373 | Staga001 | Satt504 | Satt440 | Sat_306 |
| Sat_319 | Satt373 | Sat_319 | Satg001 | Sat_279 | Sat_341 | Staga001 | Satt440 | Sat_279 | Satt388 |
| Satt545 | Sat_319 | Satt713 | Satt579 | Sat_001 | Sat_341 | Staga001 | Sat_306 | Sat_279 | Satt135 |
| Sat_227 | Satt152 |  |  |  |  |  |  |  |  |
