# Supplementary material for: QTL Location and Epistatic Effect Analysis of 100-Seed Weight Using Wild Soybean (Glycine soja Sieb. & Zucc.) Chromosome Segment Substitution Lines
Source: PLoS One. 2016 Mar 2;11(3):e0149380. doi: 10.1371/journal.pone.0149380 (PMC4774989; doi:10.1371/journal.pone.0149380)
Supplement: S5 Table — (DOCX) [file pone.0149380.s014.docx]

S5 Table The significant pairwise interactions in 2011

| 2011 |  | 2011 |  | 2011 |  | 2011 |  | 2011 |  |
| --- | --- | --- | --- | --- | --- | --- | --- | --- | --- |
| Sat_171 | Satt713 | Satt577 | Satt411 | Satt422 | Satt691 | Satt582 | Satt594 | Satt425 | Satt152 |
| Sat_171 | Satt621 | Satt577 | Sat_306 | Satt422 | Satt504 | Satt669 | Satt504 | Satt425 | Satt674 |
| Sat_319 | Satt373 | Satt577 | Satt492 | Satt422 | Satt411 | Satt672 | Sat_220 | Satt663 | Satt674 |
| Sat_319 | Satt713 | Satt577 | Satt422 | Satt422 | Sat_306 | Satt411 | Satt547 | Satt663 | Satt152 |
| Sat_232 | Satt146 | AW620774 | Sat_306 | Satt422 | Satt492 | Satt411 | Satt720 | Satt504 | Satt547 |
| Sat_232 | Satt663 | Satt565 | Satt691 | Satt422 | Satt547 | Satt411 | Satt504 | Satt504 | Satt492 |
| Sat_232 | Sat_261 | Satt565 | Satt720 | Satt422 | Satt700 | Satt411 | Sat_306 | Satt568 | Satt192 |
| Sat_232 | Satt531 | Satt565 | Satt504 | Satt531 | Sat_227 | Satt411 | Satt492 | Satt700 | Satt547 |
| Sat_232 | Sat_227 | Satt565 | Satt194 | Satt531 | Satt425 | Satt411 | Satt691 | Satt674 | Satt152 |
| Sat_232 | Satt135 | Satt565 | Satt422 | Satt531 | Satt663 | Satt691 | Satt504 | Satt596 | Satt152 |
| Sat_232 | Satt425 | Satt565 | Satt411 | Satt531 | Satt674 | Satt691 | Sat_306 | Satt547 | Satt492 |
| Sat_232 | Satt674 | Satt565 | Sat_306 | Sat_279 | Satt146 | Satt691 | Satt492 | Satt547 | Sat_306 |
| Sat_261 | Satt531 | Satt565 | Satt492 | Sat_227 | Satt135 | Satt691 | Satt547 | Satt388 | Sat_306 |
| Sat_261 | Sat_227 | Satt194 | Satt691 | Sat_227 | Satt425 | Satt720 | Satt504 | Satt678 | Satt373 |
| Sat_261 | Satt146 | Satt194 | Satt720 | Sat_227 | Satt663 | Satt720 | Sat_306 | Sat_306 | Satt492 |
| Sat_261 | Satt425 | Satt194 | Satt504 | Sat_227 | Satt674 | Satt720 | Satt492 | Satt577 | Satt504 |
| Sat_261 | Satt663 | Satt194 | Satt422 | Sat_289 | Satg001 | Satt146 | Satt504 | Satt577 | Satt194 |
| Sat_261 | Satt674 | Satt194 | Satt411 | Satt135 | Satt146 | Satt146 | Satt596 | Satt713 | Sat_289 |
| Satt197 | Satt504 | Satt194 | Satt547 | Satt135 | Satt152 | Satt146 | Satt663 | Satt713 | Satt678 |
| Sat_149 | Sat_279 | Satt194 | Sat_306 | Satt135 | Satt425 | Satt146 | Satt152 | Satt582 | Satt504 |
| Sat_149 | Satt146 | Satt194 | Satt492 | Satt135 | Satt663 | Satt146 | Satt594 | Satt582 | Satt146 |
| Satt577 | Satt691 | Satt713 | Satt373 | Satt135 | Satt674 | Satt146 | Satt425 | Satt146 | Satt674 |
| Satt425 | Satt663 |  |  |  |  |  |  |  |  |
