# Supplementary material for: QTL Location and Epistatic Effect Analysis of 100-Seed Weight Using Wild Soybean (Glycine soja Sieb. & Zucc.) Chromosome Segment Substitution Lines
Source: PLoS One. 2016 Mar 2;11(3):e0149380. doi: 10.1371/journal.pone.0149380 (PMC4774989; doi:10.1371/journal.pone.0149380)
Supplement: S1 Text — (DOCX) [file pone.0149380.s015.docx]

Annex 1 Matlab program for epistatic QTLs detected

step1: setup matlab

step2: copy test1.m-test7.m，test_key.m to the current file of matlab

step3: apply the order of matlab import the data

test1.m

genotype variant named as da, structure is cell type;

Example as follow:

'A' 'A' 'A' 'A' 'A' 'A' 'A'

'A' 'A' 'A' 'A' 'A' 'A' 'A'

'A' 'A' 'A' 'A' 'A' 'A' 'A'

'A' 'A' 'A' 'A' 'A' 'A' 'A'

'A' 'A' 'A' 'A' 'A' 'A' 'A'

'A' 'A' 'A' 'A' 'A' 'A' 'A'

'A' 'A' 'A' 'A' 'A' 'A' 'A'

'A' 'A' 'A' 'A' 'B' 'B' 'A'

'A' 'A' 'A' 'A' 'A' 'A' 'A'

'A' 'A' 'A' 'A' 'B' 'B' 'A'

test2.m

phenotype data, structure is matrix;

Example as follow:

15.9116666666667

16.0182461809274

18.1425479452055

19.1326666666667

19.7677342313112

20.0025687665425

21.1253559801179

16.7471428571429

15.1148267622461

15.3042608695652

test3.m

in the background of suinong 14, check the effect of allele gene; method were two independent sample t test.

test4.m-test7.m

the produced effect information of group was import into the matrix all, the nomenclature follow the test6.m

**test1**

[m,n]=size(da);

d=1;

for j=1:n

for k=j+1:n

for i=1:m

p{i,d}=strcat(da{i,j},da{i,k});

end

d=d+1;

end

end

**test2**

[m,n]=size(p);

for r=1:n

x(r).AA=0;

x(r).AB=0;

x(r).AC=0;

x(r).BA=0;

x(r).BB=0;

x(r).BC=0;

x(r).CA=0;

x(r).CB=0;

x(r).CC=0;

k1=1;

k2=1;

k3=1;

k4=1;

k5=1;

k6=1;

k7=1;

k8=1;

k9=1;

for i=1:m

if sum(p{i,r}=='AA')==2

x(r).AA(k1,1)=data(i);

k1=k1+1;

elseif sum(p{i,r}=='AB')==2

x(r).AB(k2,1)=data(i);

k2=k2+1;

elseif sum(p{i,r}=='AC')==2

x(r).AC(k3,1)=data(i);

k3=k3+1;

elseif sum(p{i,r}=='BA')==2

x(r).BA(k4,1)=data(i);

k4=k4+1;

elseif sum(p{i,r}=='BB')==2

x(r).BB(k5,1)=data(i);

k5=k5+1;

elseif sum(p{i,r}=='BC')==2

x(r).BC(k6,1)=data(i);

k6=k6+1;

elseif sum(p{i,r}=='CA')==2

x(r).CA(k7,1)=data(i);

k7=k7+1;

elseif sum(p{i,r}=='CB')==2

x(r).CB(k8,1)=data(i);

k8=k8+1;

elseif sum(p{i,r}=='CC')==2

x(r).CC(k9,1)=data(i);

k9=k9+1;

end

end

end

**test3**

% test the difference of phenotype with each allele gene group

[m,n]=size(p);

for r=1:n

y(r).AA=10;

y(r).AB=10;

y(r).AC=10;

y(r).BA=10;

y(r).BB=10;

y(r).BC=10;

y(r).CA=10;

y(r).CB=10;

y(r).CC=10;

if sum(x(r).AB~=0)~=0

[h,p1,ci,stats1]=ttest2(x(r).AB,x(r).AA);

y(r).AB(1)=p1;

y(r).AB(2)=stats1.tstat;

end

if sum(x(r).AC~=0)~=0

[h,p2,ci,stats2]=ttest2(x(r).AC,x(r).AA);

y(r).AC(1)=p2;

y(r).AC(2)=stats2.tstat;

end

if sum(x(r).BA~=0)~=0

[h,p3,ci,stats3]=ttest2(x(r).BA,x(r).AA);

y(r).BA(1)=p3;

y(r).BA(2)=stats3.tstat;

end

if sum(x(r).BB~=0)~=0

[h,p4,ci,stats4]=ttest2(x(r).BB,x(r).AA);

y(r).BB(1)=p4;

y(r).BB(2)=stats4.tstat;

end

if sum(x(r).BC~=0)~=0

[h,p5,ci,stats5]=ttest2(x(r).BC,x(r).AA);

y(r).BC(1)=p5;

y(r).BC(2)=stats5.tstat;

end

if sum(x(r).CA~=0)~=0

[h,p6,ci,stats6]=ttest2(x(r).CA,x(r).AA);

y(r).CA(1)=p6;

y(r).CA(2)=stats6.tstat;

end

if sum(x(r).CB~=0)~=0

[h,p7,ci,stats7]=ttest2(x(r).CB,x(r).AA);

y(r).CB(1)=p7;

y(r).CB(2)=stats7.tstat;

end

if sum(x(r).CC~=0)~=0

[h,p8,ci,stats8]=ttest2(x(r).CC,x(r).AA);

y(r).CC(1)=p8;

y(r).CC(2)=stats8.tstat;

end

end

**test4**

% generate zuhe list

[m,n]=size(da)

d=1;

for j=1:n

for k=j+1:n

id{d}(1,1)=j;

id{d}(1,2)=k;

d=d+1;

id1=id';

end

end

**test5**

%combine the final table

%id1and y combine，generate a new structure

[m,n]=size(p);

for r=1:n

z(r).id=id1{r};

z(r).AA_mean=mean(x(r).AA);

z(r).AA_num=length(x(r).AA);

z(r).AB_AA=y(r).AB;

z(r).AB_mean=mean(x(r).AB);

z(r).AB_num=length(x(r).AB);

z(r).AC_AA=y(r).AC;

z(r).AC_mean=mean(x(r).AC);

z(r).AC_num=length(x(r).AC);

z(r).BA_AA=y(r).BA;

z(r).BA_mean=mean(x(r).BA);

z(r).BA_num=length(x(r).BA);

z(r).BB_AA=y(r).BB;

z(r).BB_mean=mean(x(r).BB);

z(r).BB_num=length(x(r).BB);

z(r).BC_AA=y(r).BC;

z(r).BC_mean=mean(x(r).BC);

z(r).BC_num=length(x(r).BC);

z(r).CA_AA=y(r).CA;

z(r).CA_mean=mean(x(r).CA);

z(r).CA_num=length(x(r).CA);

z(r).CB_AA=y(r).CB;

z(r).CB_mean=mean(x(r).CB);

z(r).CB_num=length(x(r).CB);

z(r).CC_AA=y(r).CC;

z(r).CC_mean=mean(x(r).CC);

z(r).CC_num=length(x(r).CC);

end

**test6**

%control for z

s=length(z);

for i=1:s

all(i,1)=z(i).id(1);

all(i,2)=z(i).id(2);

all(i,3)=z(i).AA_mean;

all(i,4)=z(i).AA_num;

all(i,5)=z(i).AB_AA(1);

all(i,6)=z(i).AB_mean;

all(i,7)=z(i).AB_num;

all(i,8)=z(i).AC_AA(1);

all(i,9)=z(i).AC_mean;

all(i,10)=z(i).AC_num;

all(i,11)=z(i).BA_AA(1);

all(i,12)=z(i).BA_mean;

all(i,13)=z(i).BA_num;

all(i,14)=z(i).BB_AA(1);

all(i,15)=z(i).BB_mean;

all(i,16)=z(i).BB_num;

all(i,17)=z(i).BC_AA(1);

all(i,18)=z(i).BC_mean;

all(i,19)=z(i).BC_num;

all(i,20)=z(i).CA_AA(1);

all(i,21)=z(i).CA_mean;

all(i,22)=z(i).CA_num;

all(i,23)=z(i).CB_AA(1);

all(i,24)=z(i).CB_mean;

all(i,25)=z(i).CB_num;

all(i,26)=z(i).CC_AA(1);

all(i,27)=z(i).CC_mean;

all(i,28)=z(i).CC_num;

end

**test7**

k=1;

u0=41.34;

for i=1:7260

if all(i,14)<0.01

u=(z(i).AB_mean+z(i).BA_mean);

[h,p_value(k,1)]=ttest(x(i).BB+u0,u);

all_BB_BA_AB_back(k,1)=all(i,1);

all_BB_BA_AB_back(k,2)=all(i,2);

all_BB_BA_AB_back(k,3)=p_value(k,1)

k=k+1;

end

end
